# Short and equal vascular stump length after standardized laparoscopic and open surgery with central lymphadenectomy for right-sided colon cancer

**DOI:** 10.1093/bjs/znad410

**Published:** 2023-12-08

**Authors:** Kristin B Lygre, Geir E Eide, Marjolein H Liedenbaum, Idun M B Augland, Ingfrid S Haldorsen, Frank Pfeffer

**Affiliations:** Department of Gastrointestinal Surgery, Haraldsplass Deaconess Hospital, Bergen, Norway; Department of Gastrointestinal Surgery, Haukeland University Hospital, Bergen, Norway; Department of Clinical Medicine, University of Bergen, Bergen, Norway; Centre for Clinical Research, Haukeland University Hospital, Berge, Norway; Department of Global Public Health and Primary Care, University of Bergen, Bergen, Norway; Western Norway University of Applied Sciences, HVL, Bergen, Norway; Department of Radiology, Haukeland University Hospital, Bergen, Norway; Department of Radiology, Haraldsplass Deaconess Hospital, Bergen, Norway; Department of Clinical Medicine, University of Bergen, Bergen, Norway; Mohn Medical Imaging and Visualization Centre, Department of Radiology, Haukeland University Hospital, Bergen, Norway; Department of Gastrointestinal Surgery, Haukeland University Hospital, Bergen, Norway; Department of Clinical Medicine, University of Bergen, Bergen, Norway

Improvement of colon cancer surgery is an ongoing process worldwide^1,2^. Right-sided colon cancer (RCC) and left-sided colon cancer are considered two different diseases as they evolve from different embryological origins and differ in molecular profile and complexity of vascular anatomy. The extent of lymphadenectomy and the integrity of the mesocolon are considered crucial for an optimal oncological outcome. Lymphadenectomy is often measured in terms of lymph node yield, but is also influenced by factors other than the surgery. The extent of lymphadenectomy in RCC has been debated as no consensus or unambiguous definition of the medial border of the mesentery of the right colon exists. The most central mesocolic lymph nodes are located around the superior mesenteric artery and vein on the right side (*Fig. S1*). Up until recently, there has been no classification system for evaluating the completeness of mesocolic tissue removal. A new classification of the specimen in RCC takes both the extent of lymphadenectomy and the completeness of the mesocolon into consideration, using anatomical landmarks in the specimen^3^. Measurement of the length of the remaining vascular stump is accessible retrospectively^4^ and can constitute a valuable quality indicator and be a surrogate marker for the extent of lymphadenectomy.

The consecutive first 20 patients in each study group included in a randomized controlled trial comparing open and laparoscopic resection^5^ were investigated with measurement of the length of the remaining vascular stump of the ileocolic artery after standardized oncological right-sided colectomy with central lymphadenectomy. Vessels were sealed using metallic radiopaque clips (laparoscopic group) and suture ligation (open group). All patients underwent contrast-enhanced abdominal CT (portal venous phase), 6 months after surgery, as part of the standard surveillance programme (*supplementary materials*). The measurement was conducted retrospectively by two independent specialists in radiology from two different institutions.

Patient characteristics were similar in the two groups (*Table S1*). There was no difference in the mean(s.d.) length of the remaining vascular stump of the main tumour-feeding artery (4.1(0.8) mm (open) *versus* 4.1(0.9) mm (laparoscopic); *P* = 0.996) (*Table 1*). Bloodloss and lymph node yield did not differ in the two groups (*supplementary materials, Table S2*). Two patients with reported less central lymphadenectomy due to obesity and bleeding had a mean stump length of 15.5 and 16.8 mm respectively. Two other patients had a mean stump length of greater than or equal to 10 mm (*Table S3*).

**Table 1:** Length of the remaining vascular stump after oncological resection for 40 patients equally randomized to open D3 surgery at Haukeland University Hospital and laparoscopic complete mesocolic excision surgery at Haraldsplass Deaconess Hospital in Bergen (Norway) from September 2016 to June 2018

Observer	Length of the remaining vascular stump (mm)					
	Mean(s.e.m.)			Median (i.q.r.)		
	Open D3 surgery	Laparoscopic CME surgery	P*	Open D3 surgery	Laparoscopic CME surgery	*P*†
**1**	6.23 (1.57)	3.58 (0.82)	0.142	3.5 (3.8)	2.5 (3.3)	0.038
Observation 1	5.95 (1.73)	3.50 (0.82)	0.208	4.0 (4.0)	2.0 (3.0)	0.117
Observation 2	6.50 (1.45)	3.65 (0.83)	0.095	4.0 (4.0)	3.0 (4.0)	0.030
**2**	1.98 (0.54)	4.66 (1.06)	0.030	1.0 (4.0)	3.3 (4.3)	0.026
Observation 1	0.95 (0.44)	4.47 (1.09)	0.005	0.0 (0.0)	3.0 (5.0)	<0.001
Observation 2	3.00 (0.86)	4.85 (1.06)	0.183	0.5 (6.0)	3.5 (5.0)	0.114
Observer 1−observer 2	4.25 (1.79)	−1.09 (0.68)	0.008	2.8 (5.0)	−0.8 (2.3)	0.003
Total	4.10 (0.76)	4.11 (0.89)	0.996	3.9 (3.1)	3.0 (3.3)	0.440

*Gosset’s unpaired *t* test. †Wilcoxon–Mann–Whitney test. i.q.r., interquartile range; CME, complete mesocolic excision; Observer 1−observer 2, difference between observer 1 and observer 2.

Intra- and inter-observer reproducibility was investigated. Intra-observer reproducibility was good, with interclass correlation of 0.97 (observer 1) and 0.86 (observer 2). Inter-observer reproducibility was poor, with interclass correlation of 0.24 (observation 1) and 0.12 (observation 2). The main discrepancies occurred in the open group (*Table 1*).

The present study confirmed a short mean length of the remaining vascular stump, in accordance with the aim for central lymphadenectomy and vascular ligation (*Fig. S2*). Measurement of the vessel stump for the open group was demanding due to the lack of a marker on the vessel stump. Surgical quality is difficult to measure. Measurement of the remaining tumour-feeding vessel stump after resection reflects the extent of lymphadenectomy and the potential amount of residual mesentery better than other parameters. Together with evaluation of the specimen and photography of the resection area, stump length can constitute a valuable quality indicator in oncological colon surgery.

There was no difference in the length of the remaining main tumour-feeding artery after oncological RCC surgery for patients operated on with standardized open and laparoscopic right-sided colectomy with central lymphadenectomy. The remaining vessel stump was short (mean length of 4.1 mm).

## Supplementary Material

znad410_Supplementary_Data

## Data Availability

The authors confirm that the data supporting the findings of this study are available within the article and its *Supplementary material*.

## References

[1] Hohenberger W , Weber K, Matzel K, Papadopoulos T, Merkel S. Standardized surgery for colonic cancer: complete mesocolic excision and central ligation—technical notes and outcome. Colorectal Dis 2009;11:354–364; discussion 64–65 19016817 10.1111/j.1463-1318.2008.01735.x

[2] Japanese Research Society for Cancer of the Colon and Rectum . General rules for clinical and pathological studies on cancer of the colon, rectum and anus. Part I. Clinical classification. Jpn J Surg 1983;13:557–573 6672390 10.1007/BF02469505

[3] Benz S , Tannapfel A, Tam Y, Grunenwald A, Vollmer S, Stricker I. Proposal of a new classification system for complete mesocolic excison in right-sided colon cancer. Tech Coloproctol 2019;23:251–257 30838463 10.1007/s10151-019-01949-4

[4] Munkedal DLE , Rosenkilde M, West NP, Laurberg S. Routine CT scan one year after surgery can be used to estimate the level of central ligation in colon cancer surgery. Acta Oncol 2019;58:469–471 30700192 10.1080/0284186X.2019.1566770

[5] Lygre KB , Eide GE, Forsmo HM, Dicko A, Storli KE, Pfeffer F. Complications after open and laparoscopic right-sided colectomy with central lymphadenectomy for colon cancer: randomized controlled trial. BJS Open 2023;7:zrad074 37643373 10.1093/bjsopen/zrad074 PMC10465081

