# Supplementary material for: Short and equal vascular stump length after standardized laparoscopic and open surgery with central lymphadenectomy for right-sided colon cancer
Source: Br J Surg. 2023 Dec 8;111(1):znad410. doi: 10.1093/bjs/znad410 (PMC10771266; doi:10.1093/bjs/znad410)
Supplement: znad410_Supplementary_Data [file znad410_supplementary_data.docx]

**Short and equal vascular stump length after standardized laparoscopic and open surgery with central lymphadenectomy for right sided colon cancer**

Kristin B Lygre^1, 2, 3, #^, Geir E Eide ^4, 5, 6^, Marjolein H Liedenbaum^7^, Idun MB Augland^8^, Ingfrid S Haldorsen^3, 9^ Frank Pfeffer^2, 3^

^1^ Department of Gastrointestinal Surgery, Haraldsplass Deaconess Hospital, Bergen, Norway;

^2^ Department of Gastrointestinal Surgery, Haukeland University Hospital, Bergen, Norway;

^3^ Department of Clinical Medicine, University of Bergen, Bergen, Norway;

^4^ Centre for Clinical Research, Haukeland University Hospital, Berge, Norway;

^5^Department of Global Public Health and Primary Care, University of Bergen, Bergen, Norway;

^6^Western Norway University of Applied Sciences, Bergen, Norway;

^7^ Department of Radiology, Haukeland University Hospital, Bergen, Norway;

^8^Department of Radiology, Haraldsplass Deaconess Hospital, Bergen, Norway;

^9^Mohn Medical Imaging and Visualization Centre, Dep. of Radiology, Haukeland University Hospital, Bergen, Norway;

**Corresponding author.** Kristin Bentung Lygre, Department of Gastrointestinal Surgery, Haraldsplass Deaconess Hospital, Bergen, Norway, email: Kristin.Bentung.Lygre@haraldsplass.no

**ORCID ID** 0000-0003-4421-3385

**Supplementary Materials - Index**

| **Supplementary Methods** |  |
| --- | --- |
| Patient selection | *page 3* |
| Operative methods | *page 3* |
| CT protocol | *page 3* |
| Data collection | *page 4* |
| Statistical analysis | *page 4* |
| Ethical approval | *page 5* |
| **Supplementary Results** |  |
| Patient characteristics | *page 6* |
| Operative outcome variables | *page 6* |
| Photos | *page 6* |
| CT | *page 6* |
| Postoperative arterial stump (ileocolic artery) | *page 6* |
| **Supplementary Figures and Tables** |  |
| Supplementary Figure 1 | *page 7* |
| Supplementary Table 1 | *page 8* |
| Supplementary Table 2 | *page 9* |
| Supplementary Figure 2 | *page 10* |
| Supplementary Table 3 | *page 11* |
| **References** | *page 12* |
|  |  |

**Supplementary Methods**

*Patient selection*

The consecutive first 40 patients in the “*Open D3 right hemicolectomy compared to laparoscopic CME right hemicolectomy for right-sided colon cancer*”-study were included. The sample size was restricted due to limited radiologist resources. The inclusion started in September 2016, and the last postoperative CT was completed in April 2020. Only patients aged 18 - 85 years without previous colorectal cancer were included. Metastatic cancers were excluded, and all patients with ongoing treatment for other cancers were excluded. Six of the included and randomized study patients were excluded from the original trial (due to unrecognized metastasis (1), T0 cancer (3), failure to treat (1) and withdrawal of consent (1)) and thus also this analysis. All 40 patients underwent surgery in Bergen; 20 patients underwent surgery at Haraldsplass Deaconess Hospital with laparoscopic right colectomy and 20 patients were operated at Haukeland University Hospital with open right colectomy. Five patients in the laparoscopic group and two patients in the open group were operated with extended right colectomy.

*Operative methods*

The laparoscopic surgery was conducted with dissection along the superior mesenteric vein, and in the open group the dissection was along the left border of the superior mesenteric artery (Supplementary Figure 1) as described in the publication on short term results for the “*Open D3 right hemicolectomy compared to laparoscopic CME right hemicolectomy for right-sided colon cancer*”-study (1). Both groups aimed to divide the feeding vessels at their origin. CME-principles with dissection in the embryological plane were followed for both groups. Only three selected surgeons at each hospital performed the surgery in this project, and two colorectal surgeons were present during the vascular dissection.

*CT protocol*

All patients were diagnosed with colon cancer and therefore scanned with contrast enhanced (CE) abdominal CT prior to surgery for staging purpose. All patients in the open group had a preoperative three-dimensional vascular reconstruction CT to guide the surgeons. Patients in the laparoscopic group had a standard two-dimensional CT preoperatively. The postoperative CT, conducted in portal venous contrast phase, was used to measure the remaining vascular stump length. CT scans were performed on multi-slice CT scanners (Siemens AS+, Siemens Flash and Toshiba Prime at HUS and GE Revolution CT (GE Healthcare, Milwaukee, WI, USA) at HDS). Intravenous contrast was applied in all cases when not contraindicated. Datasets were reconstructed with a slice thickness of 1-3 mm. The remaining vascular stump length for the ileocolic artery (ICA) was measured.

*Data collection*

The clinical data with patient characteristics and postoperative complications (Clavien-Dindo classification) were registered prospectively in the “*Open D3 right hemicolectomy compared to laparoscopic CME right hemicolectomy for right-sided colon cancer*” project and additional clinical data obtained by reviewing electronic healthcare records. Peroperative blood-loss was determined by visual estimation by the staff at the operating theater. The pathological evaluation of all specimens was performed at the same department of pathology (Gade’s Institute at Haukeland University Hospital) according to the International Union Against Cancer Tumor Node Metastasis (TNM) system (seventh edition) and pathology data extracted from routine histopathology reports.

The analysis of the remaining vessel stumps was conducted in retrospect during 2020 and 2021. Initially (observer 2, observation 1) from deidentified CT scans via CDs, and later (observer 1, observation 1 &2 and observer 2, observation 2) from deidentified CT scans stored in a research picture archiving and communication system (PACS) (SECTRA UniVew Version 22.1.10.4793). The preoperative CT scans were available in the research PACS, but not for the first observations (observer 2, observation 1). Scans were available in coronal, axial and sagittal reformats, and the radiologists selected the most appropriate angles with the best display of the post resection arterial stump. All measurements were registered, and mean value was computed. Measurements were conducted from the most centrally orientated clip or the end of visible vessel along the inferior border of the resected vessel and to the right lateral border of the superior mesenteric artery. Each observer performed the measurements twice with a minimum of 6 weeks between. An additional 3D reconstruction of the vessels in SECTRA was performed in the patients where the vessel stump visualization was challenging.

*Statistical analysis*

Baseline and tumour characteristics were summarized using descriptive statistics. Operative outcome variables were compared with Gosset’s unpaired t-test, Wilcoxon-Mann-Whitney test and Chi square test. The mean postoperative remaining arterial stump length was compared using independent samples t-test (Gosset’s unpaired t-test) and reported as means and standard deviations (SD). The median postoperative remaining arterial stump length was compared using Wilcoxon-Mann-Whitney test. Inter- and intraobserver variability were calculated by interclass correlation and Bland-Altman plot. All statistical analyses were performed using SPSS version 26.0.0.1.

*Ethical approval*

The study is approved by the regional committee for research ethics (REK 2015/2396) and is in accordance with the “WMA Declaration of Helsinki - Ethical Principles for Medical Research Involving Human Subjects”. All patients provided written informed consent. ClinicalTrials.gov Identifier: NCT03776591.

**Supplementary Results**

*Patient characteristics*

The patients included were 19 men and 21 women (Supplementary Table 1). The groups were equal in size (n = 20).

*Operative outcome variables*

The number of lymph nodes was mean (95 % CI) 32.0 (23.2, 39.9) vs 28.5 (23.7, 34.0) open vs lap respectively p= 0.447. The two treatment groups exhibited similar bleeding (in ml) and rate of transfusion or infusion of intravenous iron. (Supplementary table 2).

*Photos*

Photos of the postoperative situs (Supplementary Figure 2) were available in 11/20 patients in the open group and 14/20 patients in the laparoscopic group. Photos were available only in two patients with measurements of vessel stump length > 10 mm.

*CT*

Most of the open group (18/20; 90 %) had accessible postoperative scans with slice thickness 1 mm and the majority of the laparoscopic group (16/20; 80 %) with slice thickness 3 mm.

*Postoperative arterial stump (ileocolic artery)*

The measurements varied from 0 to 36 mm, and 18/160 measurements were ≥ 10 mm. The difference in the measurements for observer 1 minus 2 in the two treatment groups differed significantly (4.25 mm ± 0.8 open vs 1.09 mm ± 0.7 lap; p = 0.008). (Table 1)

**Supplementary Figures and Tables**

**Supplementary figure 1** Schematic overview of the mesocolon with N1-N3 areas and corresponding Dissection level market out. Illustration of the extent of dissection of the laparoscopic CME- and open D3 approach, respectively.


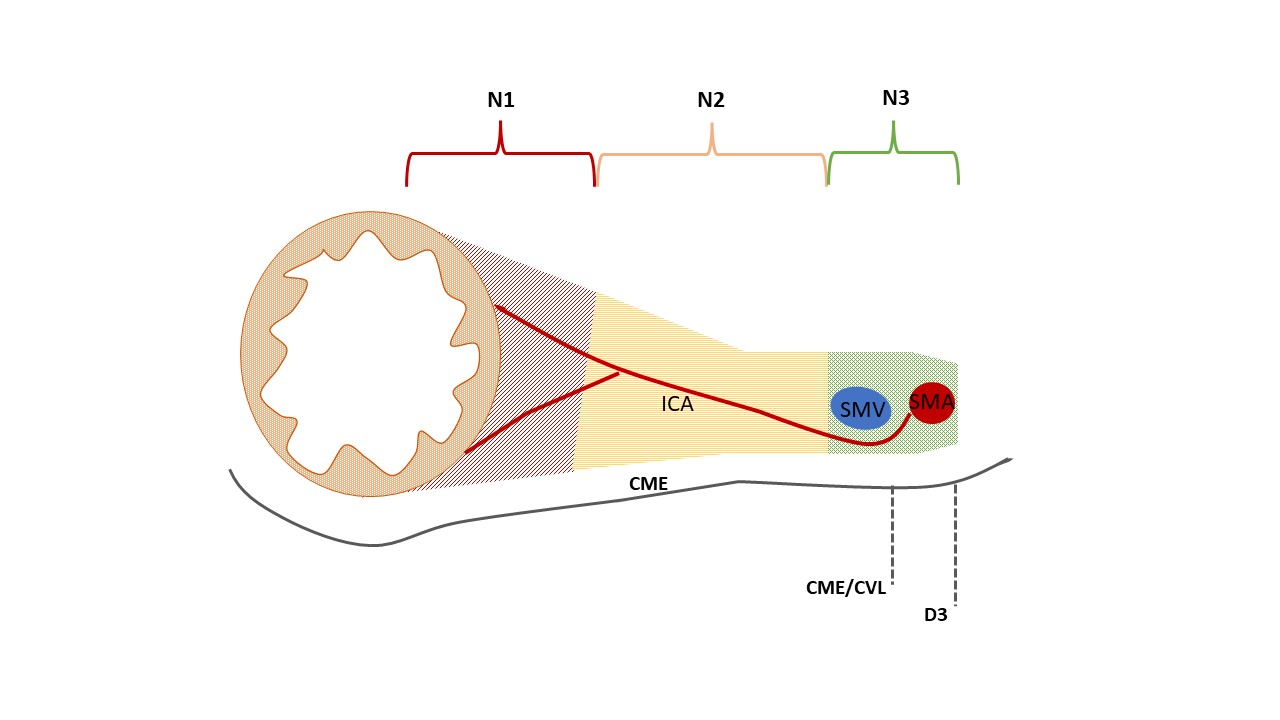


**Supplementary table 1** Basic characteristics for 40 patients equally randomised to open surgery at Haukeland University Hospital and laparoscopic surgery at Haraldsplass Deaconess Hospital, respectively, in Bergen Norway from September 2016 to June 2018.

|  | **Treatment group** | |  |
| --- | --- | --- | --- |
| **Variable**  **Category** | **Open D3**  **(n = 20)** | **Laparoscopic CME**  **(n = 20)** | **p-value** |
| Gender, *n* |  |  | 0.342^a)^ |
| Male  Female | 11  9 | 8  12 |  |
| Age (years), *mean, 95% CI* | 70.3 (66.5, 74.0) | 70.9 (66.9, 74.8) | 0.819^b)^ |
| BMI (kg/m^2^), *mean, 95% CI* | 25.7 (24.6, 26.7) | 25.8 (24.0, 27.6) | 0.893^b)^ |
| Tumour location, *n* |  |  | 0.413^a)^ |
| Coecum  Ascending  Hepatic flexure  Transverse colon | 9  10  1  0 | 9  7  2  2 |  |
| Procedure, *n* |  |  | 0.212^a)^ |
| Right colectomy  Extended right colectomy | 18  2 | 15  5 |  |

*Abbreviations:* CME = complete mesocolic excision; CI = confidence interval; BMI = body mass index

a) Pearson’s chi-square test; b) Gosset’s unpaired t-test (ref. Student 1908)

**Supplementary table 2** Per and post-operative results for 40 patients equally randomised to open surgery at Haukeland University Hospital and laparoscopic surgery at Haraldsplass Deaconess Hospital, respectively, in Bergen (Norway) from September 2016 to June 2018.

|  | **Treatment group** | |  |
| --- | --- | --- | --- |
| **Characteristic**  **Category** | **Open D3**  **(n = 20)** | **Laparoscopic CME**  **(n = 20)** | **p-value** |
| Operating time (minutes), *mean, 95% CI* | 133.5, (124.3,142.6) | 213.4, (195.0,231.8) | < 0.001^a^ |
| Blood loss (ml), *median (IQR)* | 50 (50) | 50 (68) | 0.104^b^ |
| Total lymph node yield*, mean, 95% CI* | 32.0, (25.2, 38.9) | 28.9 (23.7, 34.0) | 0.447^a^ |
| Transfusion/iron infusion, *n (%)* | 3 (15) | 2 (10) | 0.633^c^ |
| Length of stay (days*), median (IQR)* | 6 (3) | 3 (3) | <0.001^b^ |
| Recurrence, *n* | 3 | 4 | 0.677^c^ |

*Abbreviations:* CME = complete mesocolic excision; CI = confidence interval; IQR = interquartile range;

a) Gosset’s unpaired t-test; b) Wilcoxon-Mann-Whitney test; c) Chi-square test

**Supplementary figure 2** Visualisation of short vessel stump after open resection. a) Preoperative CT with visualisation of the ileocolic artery branching from the superior mesenteric artery b) Postoperative CT of the same patient with no remaining visible vessel stump of ileocolic artery c) Intraoperative photography of the vessel stump after division of the ileocolic artery in a patient operated with open resection (not the same patient as in a) and b))


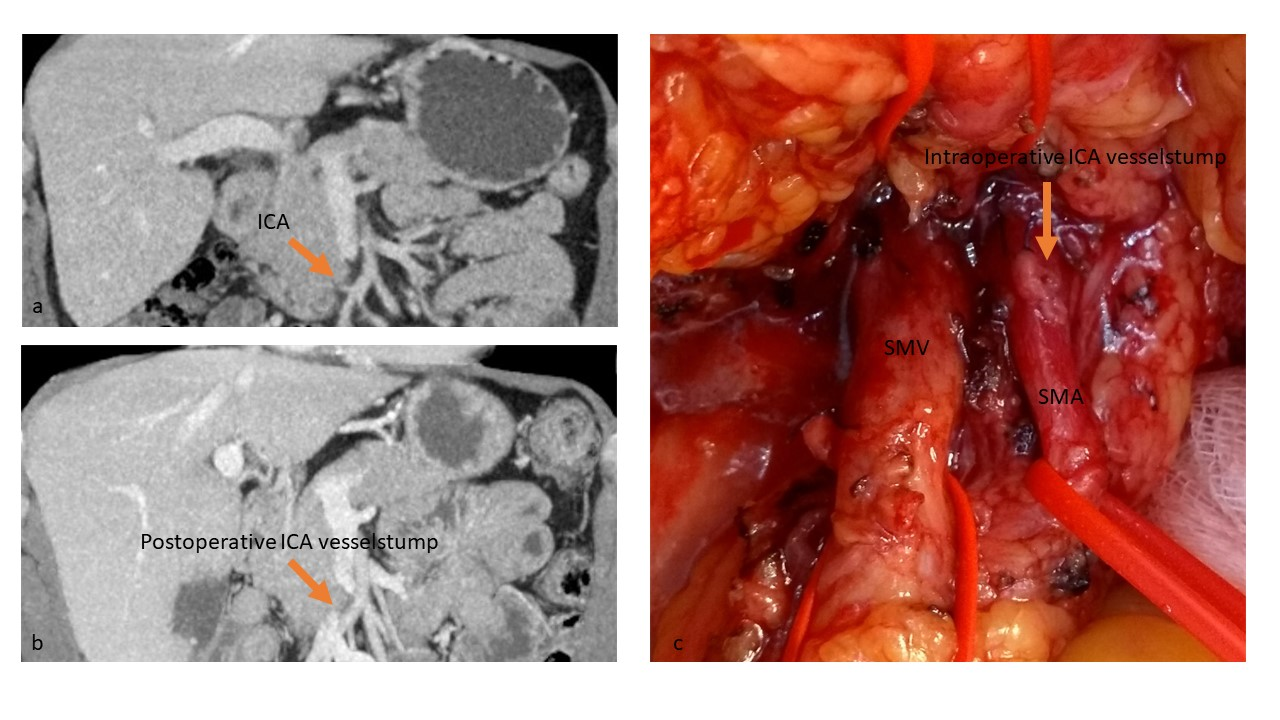


**Supplementary table 3** Measurements of the remaining vascular stump length after oncologic right-sided colectomy in 40 patients equally randomised to open surgery at Haukeland University Hospital and laparoscopic surgery at Haraldsplass Deaconess Hospital, respectively, in Bergen (Norway) from September 2016 to June 2018

| Patients | Observer 1 | | Observer 2 | | Mean length |
| --- | --- | --- | --- | --- | --- |
|  | Observation 1 | Observation 2 | Observation 1 | Observation 2 |  |
| Open |  |  |  |  |  |
|  | 4 | 4 | 0 | 0 | 2,00 |
|  | 7 | 7 | 0 | 4 | 4,50 |
|  | 2 | 3 | 0 | 0 | 1,25 |
|  | 4 | 3 | 0 | 13 | 5,00 |
|  | 6 | 12 | 0 | 1 | 4,75 |
|  | 5 | 5 | 4 | 0 | 3,50 |
|  | 2 | 4 | 0 | 0 | 1,50 |
|  | 12 | 10 | 0 | 0 | 5,50 |
|  | 2 | 4 | 0 | 5 | 2,75 |
|  | 2 | 2 | 0 | 0 | 1,00 |
|  | 4 | 3 | 0 | 0 | 1,75 |
|  | 12 | 11 | 0 | 0 | 5,75 |
|  | 3 | 3 | 0 | 3 | 2,25 |
|  | 3 | 4 | 4 | 6 | 4,25 |
|  | 3 | 2 | 0 | 0 | 1,25 |
|  | 4 | 7 | 6 | 6 | 5,75 |
|  | 6 | 6 | 0 | 5 | 4,25 |
|  | 0 | 6 | 5 | 8 | 4,75 |
|  | 36 | 31 | 0 | 0 | 16,75* |
|  | 2 | 3 | 0 | 9 | 3,50 |
| Laparoscopic |  |  |  |  |  |
|  | 2 | 2 | 0 | 1 | 1,25 |
|  | 0 | 0 | 0 | 0 | 0,00 |
|  | 6 | 6 | 9 | 9 | 7,50 |
|  | 4 | 5 | 6 | 4 | 4,75 |
|  | 15 | 15 | 17 | 15 | 15,50* |
|  | 2 | 1 | 2 | 2 | 1,75 |
|  | 3 | 3 | 3 | 3 | 3,00 |
|  | 6 | 4 | 15 | 17 | 10,50 |
|  | 2 | 2 | 3 | 4 | 2,75 |
|  | 0 | 0 | 0 | 3 | 0,75 |
|  | 4 | 4 | 4 | 5 | 4,25 |
|  | 0 | 0 | 2 | 3 | 1,25 |
|  | 3 | 4 | 1 | 0 | 1,88 |
|  | 5 | 5 | 1 | 1 | 3,00 |
|  | 2 | 2 | 6 | 6 | 4,00 |
|  | 0 | 0 | 0 | 3 | 0,75 |
|  | 2 | 3 | 5 | 5 | 3,75 |
|  | 10 | 11 | 10 | 11 | 10,50 |
|  | 2 | 3 | 4 | 4 | 3,00 |
|  | 2 | 3 | 2 | 1 | 2,00 |

^*^Patients where central lymphadenectomy was not achieved, described as D2 resection in the operation report

**References**

1. Lygre KB, Eide GE, Forsmo HM, Dicko A, Storli KE, Pfeffer F. Complications after open and laparoscopic right-sided colectomy with central lymphadenectomy for colon cancer: randomized controlled trial. BJS Open. 2023;7(4).
